# Comparing Non-invasive Prenatal Testing With Invasive Testing for the Detection of Trisomy 21

**DOI:** 10.7759/cureus.31252

**Published:** 2022-11-08

**Authors:** Rifat Mokhtar, Punit Hans, Anjana Sinha

**Affiliations:** 1 Obstetrics and Gynecology, Bhagwan Mahavir Institute of Medical Sciences, Pawapuri, IND; 2 Obstetrics and Gynecology, Patna Medical College, Patna, IND

**Keywords:** amniocentesis, non-invasive prenatal test, cell-free fetal dna, trisomy 21, trisomy, down syndrome, nipt

## Abstract

Background

Non-invasive prenatal test (NIPT) is an intermediate step between serum screening and invasive diagnostic testing. It involves analysis of the cell-free fetal DNA (cffDNA) present in the maternal blood sample for determining the likelihood of fetal aneuploidy. Owing to its high sensitivity and specificity, NIPT has quickly gained popularity across the globe since its introduction to clinical practice, making it an attractive alternative to the available screening and diagnostic tests in use. Amniocentesis is currently the gold standard test for obtaining fetal DNA and diagnosing fetal trisomy prenatally, but it is invasive and has procedure-related adverse effects. This study aims to compare NIPT and amniocentesis in pregnancies screened positive for fetal trisomy.

Material and methods

This is an analytic cross-sectional prospective study conducted in the Department of Obstetrics & Gynecology, Patna Medical College and Hospital, for two and half years from December 2018 to June 2021. A total of 34 pregnant women screened positive for trisomy 21, attending the antenatal care outpatient department, in their second trimester, with their written consent, were enrolled in the study.

Results

Out of 34 pregnant patients, three refused NIPT and directly opted for amniocentesis. A total of 31 pregnant women have undergone NIPT. A total of 28 cases were positive for trisomy 21 on both NIPT and amniocentesis. The sensitivity of NIPT was 100% with the confidence interval being 87.66% to 100.00%. The specificity of NIPT was 100% with the confidence interval being 29.24% to 100.00%.

Conclusion

The high performance and effectiveness of NIPT are undeniable. Though the process by which this test has to be integrated into the clinical practice needs more study and should be determined with meticulous assessment.

## Introduction

In 1886, J.L.H. Down, in one of his papers, described a group of intellectually disabled children with distinctive physical features [1]. Later, after 73 years, Lejeune in 1959 demonstrated that an autosomal trisomy causes Down syndrome [2]. In 95% of Down syndrome cases, trisomy 21 is found, while only 3-4% are contributed by Robertsonian translocation. It is also the most common non-lethal trisomy. According to previous studies, the approximate prevalence of Down syndrome is one in 500 recognized pregnancies [3]. As many of these pregnancies result in fetal losses and pregnancy terminations, an estimated prevalence is 13.5 in 10,000 births in the United States [3,4]. Sonography aids in screening of aneuploidy by providing accurate gestational age, detection of multifetal gestations, and identification of major and minor structural abnormalities by sonographic markers. Management of a trisomy-affected pregnancy is inclusive of the choice opted by mothers whether they want to terminate the pregnancy. It should be incorporated into the discussion of options for testing and screening. The American College of Obstetricians and Gynecologists has affirmed that aneuploidy screening should be an informed patient choice with an underlying foundation of shared decision-making that fits her clinical circumstances, values, interests, and goals [5]. Traditional or conventional screening tests do not include cell-free fetal DNA (cffDNA)-based screening. The three categories of screening are first-trimester screening, second-trimester screening, and combinations of first and second-trimester screenings. Nuchal translucency (NT) measurement by sonography is almost always included in the first-trimester screening. The first-trimester screening includes NT, pregnancy-associated plasma protein A (PAPP-A), and beta-human chorionic gonadotropin (hCG) testing, and second-trimester screening includes alpha-fetoprotein (AFP), unconjugated estriol (uE3), beta-hCG, and inhibin A testing.

Non-invasive prenatal test (NIPT) is an intermediate step between serum screening and invasive diagnostic testing. It involves analysis of the cffDNA present in the maternal blood sample for determining the likelihood of fetal aneuploidy. Owing to its high sensitivity and specificity, NIPT has quickly gained popularity across the globe since its introduction to clinical practice, making it an attractive alternative to the available screening and diagnostic tests in use. Amniocentesis is currently the gold standard test for obtaining fetal DNA and diagnosing fetal trisomy prenatally, but it is invasive and has procedure-related adverse effects.

This study aims to compare NIPT and amniocentesis in pregnancies screened positive for fetal trisomy.

## Materials and methods

This is an analytical cross-sectional prospective study conducted in the Department of Obstetrics & Gynecology, Patna Medical College and Hospital, Patna, for two and half years from December 2018 to June 2021. All the pregnant patients, reporting at the antenatal care outpatient department either after combined, fully integrated, or quadruple screening with positive reports for trisomy 21 from a standard International Organization for Standardization (ISO) 17025-accredited lab, were enrolled for the study, after taking their consent. For determining the patients to be screened positive, the specific laboratory cut-off of risk ≥ 1 in 250 for the second trimester was used [6,7]. Women with a gestational age between <10 weeks and >23 weeks, multiple gestations, conception by in vitro fertilization, history of smoking, ultrasound report showing congenital anomaly or extra-uterine pregnancy, fetal demise, fetal reduction, and weight >81 kg were excluded from the study.

Study procedure

Detailed antenatal history was taken regarding the age of the patient, last menstrual period, past and present obstetric history, both physical and mental health status of children from previous pregnancies if a patient is multiparous, history of consanguineous marriage, any child with a congenital anomaly, and mental impairment in a first-degree relative.

A general examination along with blood pressure measurement and BMI calculation was done. Per abdomen examination was done. Symphysio-fundal height was taken.

After taking the informed consent from the patient for taking NIPT, 10 mL of maternal venous blood was drawn in a streak tube and sent within six hours for centrifugation to an ISO 17025-accredited laboratory for genomic sequencing and analysis.

Amniocentesis was performed with a 20 or 22-gauge spinal needle under ultrasound guidance, with aseptic precautions. The length of the spinal needle (9 cm or longer) was decided after measuring the sonographic distance from the skin to the amnionic pocket. A pocket of amnionic fluid closed to the midline was identified by sonography, and the needle was inserted perpendicular to the skin and guided into the deepest portion of the pocket, avoiding the umbilical cord and fetal parts. The amniocytes retrieved were sent for culture, followed by karyotyping and genetic analysis.

The study was approved by the Institutional Review Board, Patna Medical College and Hospital, Patna (Letter No.: OBG/1953; dated: 6/12/2018).

## Results

Out of 34 pregnant patients, two refused NIPT and directly opted for amniocentesis, while one patient with multiple gestations was excluded. A total of 31 pregnant women have undergone NIPT. The age range of positively screened patients was from 29 years to 40 years and the range of weight was from 44 kg to 66 kg, while the minimum BMI was 17.2 kg/m^2^ and the maximum BMI was 27.8 kg/m^2^. A total of 28 cases were positive for trisomy 21 on both NIPT and amniocentesis. Two cases who previously refused NIPT were also positive for trisomy 21 on amniocentesis. Furthermore, the three cases that were negative on NIPT turned out to be negative on amniocentesis as well. The sensitivity of NIPT was 100% with the confidence interval being 87.66% to 100.00%. The specificity of NIPT was 100% with the confidence interval being 29.24% to 100.00%. There is a very strong positive correlation between the results of NIPT and amniocentesis, with the Pearson correlation coefficient being 1.00, with a p-value of <0.001. The positive predictive value of NIPT was 100%. Table 1 shows the results of NIPT and amniocentesis and Table 2 shows the calculation of sensitivity and specificity. Table 3 shows the result of the calculation for the sensitivity and specificity of NIPT in comparison with amniocentesis. Table 4 shows the correlation between NIPT and amniocentesis.

**Table 1:** Test results of NIPT and amniocentesis NIPT: non-invasive prenatal test.

Procedures	Positive result	Negative result
NIPT	28	3
Amniocentesis	30	3

**Table 2:** Calculating sensitivity and specificity NIPT: non-invasive prenatal test.

Calculation of sensitivity and specificity		
Results of NIPT	Positive	Negative
Tally with amniocentesis	28 (true positive)	3 (true negative)
Not tally with amniocentesis	0 (false positive)	0 (false negative)
Sensitivity = true positive/false negative + true positive specificity = true negative/false positive + true negative		

**Table 3:** Sensitivity and specificity of NIPT NIPT: non-invasive prenatal test.

Sensitivity and specificity of NIPT for detection of trisomy 21	
Sensitivity	100% with 95% confidence interval (87.66% to 100.00%)
Specificity	100% with 95% confidence interval (29.24% to 100.00%)

**Table 4:** Correlation between NIPT and amniocentesis NIPT: non-invasive prenatal test.

Correlation between NIPT and amniocentesis	
Pearson correlation coefficient	P-value
1.00	<0.001

## Discussion

The specificity and sensitivity of NIPT in our study were found to be similar to the previous studies [8-11], and in addition to this, these studies also reported a sensitivity of 100% and specificity of 100% for the detection of trisomy 18 and 13 by NIPT.

In a study conducted in 2016 [12], 442 maternal samples were tested and studied by the IONA® (Premaitha Health, Manchester, UK) test (a type of NIPT) with a detection rate of 100% for trisomy 21 (n = 43; 95% CI: 87.98-100%), trisomy 18 (n = 10; 95% CI: 58.72-100%), and trisomy 13 (n = 5; 95% CI: 35.88-100%), with cut-offs applied to likelihood ratio (cut-off > 1 considered high risk for trisomy) and probability risk score incorporating adjustment for maternal age (cut-off ≥ 1/150 considered high risk for trisomy). For trisomy 21, a false positive rate of 0.3% was observed for the likelihood ratio, but with adjustment for maternal age, it became 0% [12].

A meta-analysis of 2012 articles in 2016 [13] revealed the positive predictive value for trisomy 21 as 91%.

Most of the studies showed the sensitivity and specificity of NIPT for trisomy 21 were very high, while the range of positive predictive value was from 40% to 100%. This variability can be explained by the prevalence of the condition on which positive predictive value is dependent. As the prevalence of fetal trisomy is very low, NIPT is very sensitive to slight changes in the number of true positives and false positive rates if applied to the general population. But for a high-risk population, as in our study, positive predictive value can turn out to be as high as 100%.

In this study, pregnant women with multiple gestations were excluded as cffDNA per fetus is low; however, the American College of Obstetricians and Gynecologists [14] and the International Society for Prenatal Diagnosis [15] have recommended NIPT for screening common trisomies in twin pregnancies. There are not many studies on triplets.

Patients weighing > 81 kg were excluded from the study, though the definition of adequate cffDNA is 4% of all cell-free DNA in maternal blood, which is independent of maternal BMI. In obese pregnant patients, the cffDNA fraction is reduced in maternal blood because of dilution in the larger maternal blood volume and an increased contribution of maternal cell-free DNA from the adipose tissue apoptosis [16]. Postponing NIPT, in patients with BMI ≥ 35 kg/m^2^, to an older gestational age would not help in reducing test failures because the fetal cell-free DNA rises more slowly in obese patients [17,18].

Almost all the screening and diagnostic tests were completed around 22 to 23 weeks to give patients ample safe time for termination of an affected pregnancy.

Limitations of the study include a single-center study and a small sample size.

## Conclusions

Patients who underwent NIPT showed their strong support for the test as it was non-invasive and had the advantage of earlier detection with more accurate results than the traditional screening. In the present study, NIPT was as accurate as amniocentesis for prenatal detection of trisomy 21 in positively screened patients. Thus, potential risks of procedure-related miscarriages of invasive testing can be reduced with the introduction of NIPT. Though the process by which this test has to be integrated into the practical environment needs more study and should be determined with meticulous assessment.
